# Gender-Based Differences and Associated Factors Surrounding Excessive Smartphone Use Among Adolescents: Cross-sectional Study

**DOI:** 10.2196/30889

**Published:** 2021-11-22

**Authors:** Emma Claesdotter-Knutsson, Frida André, Maria Fridh, Carl Delfin, Anders Hakansson, Martin Lindström

**Affiliations:** 1 Child and Adolescent Psychiatry, Department of Clinical Sciences Lund Faculty of Medicine Lund University Lund Sweden; 2 Department of Clinical Sciences Lund Faculty of Medicine Lund University Lund Sweden; 3 Social Medicine and Health Policy Department of Clinical Sciences in Malmö Lund University Malmo Sweden; 4 Psychiatry, Malmö Addiction Centre, Gambling Disorder Unit Department of Clinical Sciences Lund Faculty of Medicine, Lund University Lund Sweden; 5 Social Medicine and Health Policy, Centre for Primary Health Care Research Department of Clinical Sciences in Malmö Lund University Malmo Sweden

**Keywords:** smartphone, cell phone, adolescent, sleep, anxiety, substance use, nicotine, alcohol drinking, smartphone use, addiction, behavioral addiction, worry, pathology, internet

## Abstract

**Background:**

Excessive smartphone use is a new and debated phenomenon frequently mentioned in the context of behavioral addiction, showing both shared and distinct traits when compared to pathological gaming and gambling.

**Objective:**

The aim of this study is to describe excessive smartphone use and associated factors among adolescents, focusing on comparisons between boys and girls.

**Methods:**

This study was based on data collected through a large-scale public health survey distributed in 2016 to pupils in the 9th grade of primary school and those in the 2nd grade of secondary school. Bayesian binomial regression models, with weakly informative priors, were used to examine whether the frequency of associated factors differed between those who reported excessive smartphone use and those who did not.

**Results:**

The overall response rate was 77% (9143/11,868) among 9th grade pupils and 73.4% (7949/10,832) among 2nd grade pupils, resulting in a total of 17,092 responses. Based on the estimated median absolute percentage differences, along with associated odds ratios, we found that excessive smartphone use was associated with the use of cigarettes, alcohol, and other substances. The reporting of anxiety and worry along with feeling low more than once a week consistently increased the odds of excessive smartphone use among girls, whereas anxiety and worry elevated the odds of excessive smartphone use among boys. The reporting of less than 7 hours of sleep per night was associated with excessive smartphone use in all 4 study groups.

**Conclusions:**

The results varied across gender and grade in terms of robustness and the size of estimated difference. However, excessive smartphone use was associated with a higher frequency of multiple suspected associated factors, including ever having tried smoking, alcohol, or other substances; poor sleep; and often feeling low and feeling anxious. This study sheds light on some features and distinctions of a potentially problematic behavior among adolescents.

## Introduction

Smartphones are the preferred tools for web-based activity, and regardless of age, almost every person possesses a smartphone [1,2]. Adolescence is a very sensitive period, wherein many physiological, psychological, and social changes occur, making this age group vulnerable to potential adverse effects of cellphone use, including depressive symptoms, anxiety, and low self-esteem [1,2]. Smartphone use is a new and debated phenomenon frequently mentioned in the context of behavioral addiction, demonstrating both common and distinct traits when compared to pathological gaming and gambling among adolescents [3-5].

Research on problematic or addictive smartphone use has expanded during the last decade, as the proportion of smartphone users has steadily increased [6-9]. Excessive smartphone use is characterized by maladaptive smartphone use with functional impairment. Excessive smartphone use may lead to symptoms commonly observed in substance use disorders, such as tolerance, withdrawal after periods of nonuse, continued use despite adverse effects, and difficulty controlling use [10,11]. Moreover, overuse of smartphones has been associated with increased anxiety, depression, poor sleep quality, low self-esteem, and higher perceived stress, as well as other addictions such as addiction to alcohol tobacco and illicit drugs [12-14].

Unlike both gaming and gambling, excessive use of smartphones appear to be more common among girls, and the motives for smartphone use seemingly show gender-based differences [15,16]. Boys are more likely to use their phones for gaming, media sharing, and internet searches, whereas girls are more likely to use their phones for social reasons—social media or texting [15,16]. Researchers have suggested different problems correlating to different motives for smartphone use [17].

Given the increasing interest of behavioral addictions and alarming reports on the consequences of screen time and adolescents increasing psychological complaints [3,5,6], this study aims to address knowledge gaps concerning the frequency of excessive smartphone use among Swedish adolescents, and whether the prevalence of suspected associated factors differed between those who reported excessive smartphone use and those who did not. Specifically, we used a large sample of Swedish pupils from primary and secondary schools to investigate whether differences existed between the two groups in terms of the following outcomes: (1) often feeling low; (2) often feeling anxious; (3) self-reported attention deficit hyperactive disorder (ADHD); (4) self-reported autism spectrum disorder (ASD); (5) being satisfied with one’s own general health; (6) poor sleep; (7) loneliness; and having tried (8) smoking, (9) alcohol, and (10) other substances.

## Methods

### Participants and Procedures

Data were collected from a public health survey distributed in 2016 to pupils in the 9th grade of primary school and 2nd grade of secondary school. The survey distribution covered all 33 municipalities in Skåne, a region in southern Sweden. The overall response rate was 77% (9143/11,868) among 9th grade pupils and 73.4% (7949/10,832) among 2nd grade pupils, resulting in a total of 17,092 responses. The main purpose of the survey was to investigate health and various social factors among Swedish adolescents. Previous school surveys in Skåne were primarily focused on alcohol, drug, and tobacco use. In contrast, the public health school survey of 2016 included a broad spectrum of public health questions regarding demographics and family characteristics (section A); general self-perceived health (section B); accidents and injuries (section C); leisure-time activities and habits (section D); dietary habits (section E); alcohol (section F); tobacco smoking and snuff use (section G); narcotic drugs (section H); sex and life together (section I); school context (section J); security and exposure (section K); gambling (section L); and general health, life satisfaction, and beliefs concerning the future (section M).

The survey was provided by the regional council of Scania County (Region Skåne) in cooperation with the municipal association of Skåne, and it was answered anonymously in classroom settings. Participation was voluntary; all questions were described as optional, and all measures were based on self-reports (see Multimedia Appendix 1).

### Measures

#### Excessive Smartphone Use

The survey contained a 6-item questionnaire about mobile phone habits that has been previously used in a large-scale European study called “Net Children Go Mobile” [18]. The questionnaire begins with asking the respondents “in the past 12 months, how often have these things happened to you?” and then proceeds to list the following 6 statements: (1) “I have felt bothered when I could not check my mobile phone”; (2) “I have caught myself doing things on my mobile phone that I was not really interested in”; (3) “I have felt a strong need to check my mobile phone to see if anything new has happened”; (4) “I have spent less time than I should with either family, friends or doing schoolwork”; (5) “I find myself using my mobile phone even in places/situations where it is not appropriate”; and (6) “I have tried unsuccessfully to spend less time using my mobile phone.” Respondents were asked to state the degree to which they agreed with each statement using a 5-point scale (“very often,” “fairly often,” “not very often,” “almost never,” or “never”). We created a new binary variable labeled “Excessive smartphone use.” Respondents who answered “often” or “very often” to 2 or more of the 6 statements were categorized as “yes,” and all others were categorized as “no” [18].

#### Associated Factors

Based on previous research outlined in the Introduction, combined with clinical experience, we chose to investigate a broad range of suspected associated factors. These factors were related to overall well-being, mental health, and various risk-taking or adverse behaviors. Using the available survey data, we created 9 new, binary variables in order to examine the frequency of each factor: (1) often feeling low, (2) often feeling anxious, (3) ADHD, (4) ASD, (5) loneliness, (6) poor sleep, (7) tried smoking, (8) tried alcohol, and (9) tried other substances.

Respondents’ psychological health was assessed using 2 questions from the Health Behaviour in School-Aged Children Symptom Checklist, both with separately verified and satisfactory test-retest reliability [19]. Specifically, respondents rated, on a 5-point scale (“about every day,” “more than once a week,” “about every week,” “about every month,” or “rarely or never”), how often they had felt low and felt anxious or worried during the past 6 months. We created 2 new binary variables, labeled “often feeling low” and “often feeling anxious,” where those who answered “about every day” or “more than once a week” were categorized as “yes,” and all others were categorized as “no.”

Questions about long-term somatic or psychiatric disorders were also included in the survey. Respondents were asked whether they had ADHD or attention deficit disorder (ADD) and autism or Asperger syndrome, and based on their answers (ie, “yes” or “no”), 2 new binary variables—labeled “ADHD” and “ASD”—were created. Respondents who affirmed ADHD/ADD or ASD were categorized as “yes,” and all others were categorized as “no.”

Further, respondents rated, on a 4-point scale (“have no close friend,” “have one close friend,” “have two close friends,” or “have several close friends”), whether they presently have a close friend with whom they could talk in confidence about almost any personal matter. We created a new binary variable, labeled “loneliness,” with those answering “have no close friend” classified as “yes,” and all others classified as “no.”

Next, respondents were asked, “How would you describe your health in general?” with 5 possible response options (“very good,” “good,” “fairly good,” “bad,” or “very bad”). A new binary variable, labeled “satisfied with your own geneal health,” was created, with the answers “very good” and “good” categorized as “yes,” and all other answers categorized as “no.”

Thereafter, respondents rated, on a 3-point scale, how many hours a night they usually sleep on weekdays (“less than 7 hours,” “7-9 hours,” or “more than 9 hours”). Based on their responses, we created a new binary variable, labeled “poor sleep,” with those answering “less than 7 hours” classified as “yes,” and all others classified as “no”.

The survey also included questions about smoking, alcohol consumption, and other substance use. For smoking, respondents were asked whether they smoke cigarettes, and their answers were recorded on a 7-point scale (“no, I have never smoked”; “no, but I have tried”; “no, I have smoked but have since quit”; “yes, when I’m on a party”; “yes, sometimes”; “yes, almost every day”; or “yes, every day”). A new binary variable labeled “tried smoking” was created, with those answering “no, I have never smoked” classified as “no,” and all other responses classified as “yes.”

For alcohol habits, respondents were asked whether they had ever drunk alcohol, with possible answers being “yes” or “no.” A new binary variable labeled “tried alcohol” was created, with those answering “yes” classified as “yes,” and those answering “no” classified as “no.”

Finally, for other substance use, respondents were asked to rate, on a 4-point scale (“no”; “yes, more than 12 months ago”; “yes, during the last 12 months”; or “yes, during the last 30 days”), whether they ever had used other substances (eg, narcotics). A new binary variable labeled “tried other substances” was created, with those answering “no” classified as “no,” and all other responses classified as “yes.”

### Statistical Analysis

The R statistical programming language (version 4.0.4) [20], along with several functions from the tidyverse package [21], was used for intermediate data processing and statistical analysis. We opted for a fully Bayesian approach, and all Bayesian models were specified using the R package brms [22]. The brms package interfaces R with the Stan probabilistic programming language [23], which is a state-of-the-art language for specifying and estimating Bayesian models. Bayesian binomial regression models were used to examine whether the frequency of the associated factors outlined above differed between adolescents reporting excessive smartphone use and those who did not. All models used weakly informative priors centered around zero, which should provide moderate regularization while still having minimal impact on obtained estimates [24]. Finally, the R package emmeans [25] was used for postprocessing results.

We present group differences as estimated median absolute percentage differences along with the associated odds ratio (OR), reported with 95% highest density intervals (HDIs). In contrast to a frequentist CI, the 95% HDI may be interpreted such that it has a 95% probability of actually containing the values inside it [26]. Furthermore, the region of practical equivalence (ROPE) approach was used to determine whether an estimated difference was of practical and/or clinical importance [26]. Specifically, we considered an estimated difference of at least 5% (in either direction) as the minimal difference for “practical equivalence.” If the 95% HDI was not beyond this cutoff value, we deemed the results as *uncertain* in terms of practical and clinical importance.

## Results

### Prevalence of Excessive Smartphone Use

Information about gender was missing for 86 respondents, bringing the total sample size available for group-based analysis to 17,006. Furthermore, there were varying levels of missing data for smartphone use as well as for the associated factors, as indicated in the tables below. Excessive smartphone use was more prevalent among girls (approximately 60%) than among boys (approximately 35%) in both grades (see Table 1 for details). Although results varied across gender and grade in terms of robustness and size of the estimated differences, overall, we found that excessive smartphone use was associated with a higher frequency of multiple suspected associated factors such as ever having tried smoking, alcohol, and other substances; poor sleep; and often feeling low and often feeling anxious. Several of these findings were both robust, with differences exceeding the ROPE with 95% probability by a large margin, and substantial, with some estimated differences reaching as high as 15%. In addition, for several other variables where the differences, with 95% probability, did not exceed the ROPE, the differences nonetheless robustly exceeded zero.

**Table 1:** Frequency of excessive smartphone use among school pupils in southern Sweden, based on data collected in 2016.

Study group	Total respondents, n	Valid responses, n (%)	Excessive smartphone use, n (%)	Non-excessive smartphone use, n (%)
Boys in 9th grade of primary school	4609	4187 (90.8)	1492 (35.6)	2695 (64.4)
Girls in 9th grade of primary school	4497	4232 (94.1)	2515 (59.4)	1717 (40.6)
Boys in 2nd grade of secondary school	3945	3605 (91.4)	1342 (37.2)	2263 (62.8)
Girls in 2nd grade of secondary school	3955	3749 (94.8)	2233 (59.6)	1516 (40.4)

### Boys in the 9th Grade of Primary School

A total of 33.6% (499/1484) of the boys in the 9th grade of primary school who reported excessive smartphone use were categorized as self-reporting poor sleep compared to 25.1% (674/2682) of those who did not report excessive smartphone use, with an estimated difference of 8.5% (95% HDI 6.1%, 10.9%) and an associated OR of 1.51 (95% HDI 1.33, 1.68). Furthermore, participants who reported excessive smartphone use had a higher frequency of having tried smoking (575/1435, 40.1%) and alcohol (939/1453, 64.6%) than those who did not report excessive smartphone use (smoking: 685/2609, 26.3%; alcohol: 1378/2646, 52.1%), with an estimated difference of 13.8% (95% HDI 11.3%, 16.5%) and OR of 1.88 (95% HDI 1.66, 2.1) for smoking and an estimated difference of 12.5% (95% HDI 9.9%, 15.1%) and OR of 1.68 (95% HDI 1.5, 1.87) for alcohol use. Furthermore, boys who reported excessive smartphone use had higher frequencies of often feeling low, often feeling anxious, ASD, and having tried other substances, as well as a lower frequency of being satisfied with their own health, although these differences did not reliably exceed the ROPE.

**Figure 1:**
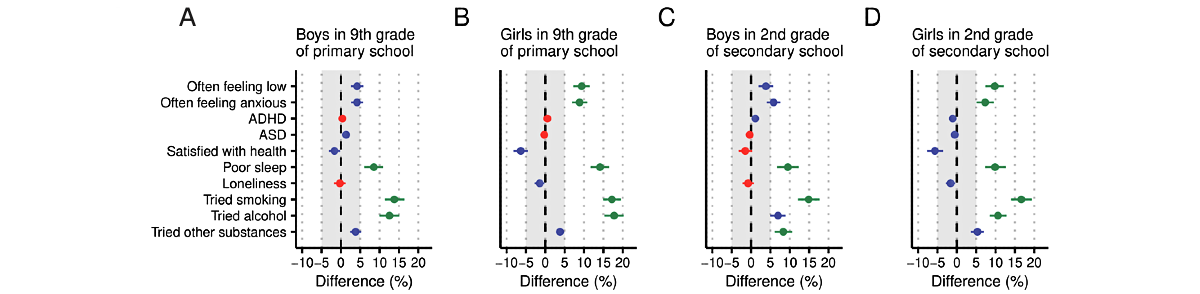
. Estimated differences in the frequency of associated factors between respondents who reported excessive smartphone use and those who did not. Dots represent posterior medians, and lines represent 95% highest density intervals. The shaded area shows the region of practical equivalence (ROPE) of ±5%. Estimated differences that, with 95% probability, are larger than the ROPE are represented in green, whereas estimated differences that, with 95% probability, are larger than zero but smaller than the ROPE are represented in blue. Differences, with 95% probability, not larger than zero are represented in red. Estimates are based on data collected among school pupils in southern Sweden in 2016.

In summary, excessive smartphone use among boys in the 9th grade of primary school was robustly associated with a higher frequency of poor sleep and having tried smoking and alcohol. Details are presented in Table 2 and Figure 1.

**Table 2:** Excessive smartphone use and associated factors among boys in the 9th grade of primary school, based on data collected in southern Sweden in 2016.

Factor^a^	Excessive smartphone use			Non-excessive smartphone use			Estimated difference (%) (95% HDI^b^)		OR^c^ (95% HDI)
	Total respondents, n	Value, n (%)	Total respondents, n		Value, n (%)				
Often feeling low (n=4053)	1442	162 (11.2)	2611		184 (7)	4.2 (2.6, 5.8)		1.67 (1.37, 1.99)	
Often feeling anxious (n=4039)	1438	143 (9.9)	2601		150 (5.8)	4.2 (2.7, 5.7)		1.8 (1.45, 2.18)	
Satisfied with health (n=3799)	1328	1228 (92.5)	2471		2326 (94.1)	–1.7 (–3.1, –0.2)		0.77 (0.6, 0.94)	
ADHD^d^ (n=4056)	1436	43 (3)	2620		68 (2.6)	0.4 (–0.5, 1.3)		1.15 (0.8, 1.55)	
ASD^e^ (n=4053)	1437	43 (3)	2616		43 (1.6)	1.3 (0.5, 2.2)		1.85 (1.21, 2.54)	
Poor sleep (n=4166)	1484	499 (33.6)	2682		674 (25.1)	*8.5 (6.1, 10.9)* ^f^		1.51 (1.33, 1.68)	
Loneliness (n=4154)	1486	126 (8.5)	2668		233 (8.7)	–0.3 (–1.8, 1.2)		0.97 (0.79, 1.16)	
Tried smoking (n=4044)	1435	575 (40.1)	2609		685 (26.3)	*13.8 (11.3, 16.5)* ^f^		1.88 (1.66, 2.1)	
Tried alcohol (n=4099)	1453	939 (64.6)	2646		1378 (52.1)	*12.5 (9.9, 15.1)* ^f^		1.68 (1.5, 1.87)	
Tried other substances (n=4004)	1408	124 (8.8)	2596		130 (5)	3.8 (2.4, 5.3)		1.83 (1.45, 2.24)	

^a^Note that the total number of respondents for each factor differs due to missing data.

^b^HDI: highest density interval.

^c^OR: odds ratio.

^d^ADHD: attention deficit hyperactivity disorder.

^e^ASD: autism spectrum disorder.

^f^Estimated differences that, with 95% probability, are above the prespecified cutoff for practical equivalence are italicized.

### Girls in the 9th Grade of Primary School

Of the girls who reported excessive smartphone use, 27.4% (678/2475) reported often feeling low and 22.9% (565/2469) reported often feeling anxious, as compared to 18% (303/1684) and 14% (236/1682), respectively, of the girls who did not report excessive smartphone use. The estimated difference and OR for often feeling low were 9.4% (95% HDI 7.2%, 11.5%) and 1.72 (95% HDI 1.5, 1.94), respectively, and the corresponding values for often feeling anxious were 8.9% (95% HDI 6.9%, 10.9%) and 1.82 (95% HDI 1.57, 2.08), respectively. In addition, 41.9% (1052/2509) of those reporting excessive smartphone use were classified as having poor sleep, compared to 27.8% (473/1702) of those not who did not report excessive smartphone use, with an estimated difference of 14.1% (95% HDI 11.7%, 16.5%) and an associated OR of 1.88 (95% HDI 1.67, 2.08).

Girls who reported excessive smartphone use also reported higher frequencies of having tried smoking (985/2478, 39.7%) and alcohol (1568/2494, 62.9%) compared to those not who did not (381/1689, 22.6% for smoking and 768/1703, 45.1%, for alcohol use), with an estimated difference of 17.2% (95% HDI 14.9%, 19.6%) and OR of 2.27 (95% HDI 2, 2.53) for smoking, and an estimated difference of 17.8% (95% HDI 15.2%, 20.3%) and OR of 2.06 (95% HDI 1.85, 2.29) for alcohol use. Moreover, girls with excessive smartphone use had lower frequencies of being satisfied with their own health as well as loneliness, and a higher frequency of having tried other substances, but these differences did not reliably exceed the ROPE.

In summary, excessive smartphone use among girls in the 9th grade of primary school was robustly associated with a higher frequency of often feeling low, often feeling anxious, poor sleep, and having tried smoking and alcohol. Details are presented in Table 3 and Figure 1.

**Table 3:** Excessive smartphone use and associated factors among girls in the 9th grade of primary school, based on data collected in southern Sweden in 2016.

Factor^a^	Excessive smartphone use			Non excessive smartphone use			Estimated difference (%) (95% HDI^b^)		OR^c^ (95% HDI)	
	Total respondents, n	Value, n (%)	Total respondents, n		Value, n (%)					
Often feeling low (n=4159)	2475	678 (27.4)	1684		303 (18)	*9.4 (7.2, 11.5)* ^d^		1.72 (1.5, 1.94)		
Often feeling anxious (n=4151)	2469	565 (22.9)	1682		236 (14)	*8.9 (6.9, 10.9)* ^d^		1.82 (1.57, 2.08)		
Satisfied with health (n=3998)	2363	1940 (82.1)	1635		1446 (88.4)	–6.3 (–8.2, –4.5)		0.6 (0.5, 0.69)		
ADHD^e^ (n=4121)	2444	80 (3.3)	1677		46 (2.7)	0.5 (–0.4, 1.4)		1.2 (0.85, 1.6)		
ASD^f^ (n=4108)	2436	24 (1)	1672		21 (1.3)	–0.3 (–0.8, 0.3)		0.78 (0.43, 1.21)		
Poor sleep (n=4211)	2509	1052 (41.9)	1702		473 (27.8)	*14.1 (11.7, 16.5)* ^d^		1.88 (1.67, 2.08)		
Loneliness (n=4210)	2507	139 (5.5)	1703		119 (7)	–1.4 (–2.7, –0.2)		0.78 (0.62, 0.95)		
Tried smoking (n=4167)	2478	985 (39.7)	1689		381 (22.6)	*17.2 (14.9, 19.6)* ^d^		2.27 (2, 2.53)		
Tried alcohol (n=4197)	2494	1568 (62.9)	1703		768 (45.1)	*17.8 (15.2, 20.3)* ^d^		2.06 (1.85, 2.29)		
Tried other substances (n=4146)	2454	150 (6.1)	1692		39 (2.3)	3.8 (2.8, 4.8)		2.78 (1.99, 3.65)		

^a^Note that the total number of respondents for each factor differs due to missing data.

^b^HDI: highest density interval.

^c^OR: odds ratio.

^d^Estimated differences that, with 95% probability, are above the prespecified cutoff for practical equivalence are italicized.

^e^ADHD: attention deficit hyperactivity disorder.

^f^ASD: autism spectrum disorder.

### Boys in the 2nd Grade of Secondary School

Boys who reported excessive smartphone use had higher frequencies of poor sleep (636/1336, 47.6% vs 858/2253, 38.1%), having tried smoking (851/1292, 65.9% vs 1120/2195, 51%), and having tried other substances (280/1267, 22.1% vs 300/2181, 13.8%) compared to those who did not report excessive smartphone use, with estimated differences of 9.5% (95% HDI 6.7%, 12.4%) and OR 1.48 (95% HDI 1.31, 1.65) for poor sleep, 14.9% (95% HDI 12.1%, 17.7%) and OR 1.85 (95% HDI 1.64, 2.08) for having tried smoking, and 8.3% (95% HDI 6.1%, 10.6%) and OR 1.78 (95% HDI 1.52, 2.06) for having tried other substances. Boys who reported excessive smartphone use also had higher frequencies of often feeling low, often feeling anxious, ASD, and having tried alcohol, although these differences did not reliably exceed the ROPE.

In summary, excessive smartphone use among boys in the 2nd grade of secondary school was robustly associated with a higher frequency of poor sleep and having tried smoking and other substances. Details are presented in Table 4 and Figure 1.

**Table 4:** Excessive smartphone use and associated factors among boys in the 2nd grade of secondary school, based on data collected in southern Sweden in 2016.

Factor^a^	Excessive smartphone use			Non excessive smartphone use			Estimated difference (95% HDI^b^)		OR^c^ (95% HDI)
	Total respondents, n	Value, n (%)	Total respondents, n		Value, n (%)				
Often feeling low (n=3519)	1312	183 (13.9)	2207		223 (10.1)	3.8 (2, 5.7)		1.44 (1.2, 1.7)	
Often feeling anxious (n=3521)	1312	172 (13.1)	2209		161 (7.3)	5.8 (4.1, 7.6)		1.92 (1.56, 2.29)	
Satisfied with health (n=3278)	1190	1069 (89.8)	2088		1907 (91.3)	–1.5 (–3.2, 0.3)		0.84 (0.68, 1.01)	
ADHD^d^ (n=3519)	1305	44 (3.4)	2214		50 (2.3)	1.1 (0.2, 2.1)		1.51 (1.01, 2.06)	
ASD^e^ (n=3516)	1308	28 (2.1)	2208		55 (2.5)	–0.4 (–1.2, 0.5)		0.85 (0.54, 1.2)	
Poor sleep (n=3589)	1336	636 (47.6)	2253		858 (38.1)	*9.5 (6.7, 12.4)* ^f^		1.48 (1.31, 1.65)	
Loneliness (n=3582)	1334	95 (7.1)	2248		177 (7.9)	–0.8 (–2.2, 0.8)		0.9 (0.71, 1.1)	
Tried smoking (n=3487)	1292	851 (65.9)	2195		1120 (51)	*14.9 (12.1, 17.7)* ^f^		1.85 (1.64, 2.08)	
Tried alcohol (n=3535)	1312	1149 (87.6)	2223		1792 (80.6)	7 (4.9, 9)		1.7 (1.43, 1.99)	
Tried other substances (n=3448)	1267	280 (22.1)	2181		300 (13.8)	*8.3 (6.1, 10.6)* ^f^		1.78 (1.52, 2.06)	

^a^Note that the total number of respondents for each factor differs due to missing data.

^b^HDI: highest density interval.

^c^OR: odds ratio.

^d^ADHD: attention deficit hyperactivity disorder.

^e^ASD: autism spectrum disorder.

^f^Estimated differences that, with 95% probability, are above the prespecified cutoff for practical equivalence are italicized.

### Girls in the 2nd Grade of Secondary School

Girls who reported excessive smartphone use had had higher frequencies of often feeling low (702/2198, 31.9% vs 332/1499, 22.1%) and often feeling anxious (560/2211, 25.3% vs 269/1493, 18%), with an estimated difference of 9.8% (95% HDI 7.3%, 12.1%) and OR of 1.65 (95% HDI 1.44, 1.86) for often feeling low and an estimated difference of 7.3% (95% HDI 5.1%, 9.5%) and OR of 1.55 (95% HDI 1.34, 1.76) often feeling anxious. In addition, 48.6% (1081/2224) of girls who reported excessive smartphone use were classified as having poor sleep, compared to 38.7% (584/1508) of those who did not report, with an estimated difference of 9.9% (95% HDI 7.3%, 12.7%) and an associated OR of 1.5 (95% HDI 1.33, 1.67).

Furthermore, girls who reported excessive smartphone use had higher frequencies of having tried smoking (1299/2181, 59.6% vs 640/1492, 42.9%) and alcohol (1903/2201, 86.5% vs 1136/1498, 75.8%), with an estimated difference of 16.7% (95% HDI 14%, 19.4%) and OR of 1.96 (95% HDI 1.74, 2.18) for smoking and an estimated difference of 10.6% (95% HDI 8.5%, 12.8%) and OR of 2.04 (95% HDI 1.75, 2.33) for alcohol. Finally, although the differences did not reliably exceed the ROPE, girls who reported excessive smartphone use had a relatively higher frequency of having tried other substances, as well as lower frequencies of ADHD, ASD, being satisfied with one’s own health, and loneliness.

In summary, excessive smartphone use among girls in the 2nd grade of secondary school was robustly associated with a higher frequency of often feeling low, often feeling anxious, poor sleep, and having tried smoking and alcohol. Details are presented in Table 5 and Figure 1.

**Table 5:** Excessive smartphone use and associated factors among girls in the 2nd grade of secondary school, based on data collected in southern Sweden in 2016.

Factor^a^	Excessive smartphone use			Non-excessive smartphone use			Estimated difference (%) (95% HDI^b^)		OR^c^ (95% HDI)
	Total respondents, n	Value, n (%)	Total respondents, n		Value, n (%)				
Often feeling low (n=3697)	2198	702 (31.9)	1499		332 (22.1)	*9.8 (7.3, 12.1)* ^d^		1.65 (1.44, 1.86)	
Often feeling anxious (n=3704)	2211	560 (25.3)	1493		269 (18)	*7.3 (5.1, 9.5)* ^d^		1.55 (1.34, 1.76)	
Satisfied with health (n=3488)	2064	1650 (79.9)	1424		1219 (85.6)	–5.7 (–7.7, –3.5)		0.67 (0.57, 0.78)	
ADHD^e^ (n=3682)	2185	61 (2.8)	1497		58 (3.9)	–1.1 (–2.1, –0.1)		0.71 (0.5, 0.94)	
ASD^f^ (n=3679)	2184	13 (0.6)	1495		17 (1.1)	–0.5 (–1.1, 0)		0.52 (0.24, 0.88)	
Poor sleep (n=3732)	2224	1081 (48.6)	1508		584 (38.7)	*9.9 (7.3, 12.7)* ^d^		1.5 (1.33, 1.67)	
Loneliness (n=3741)	2227	98 (4.4)	1514		91 (6)	–1.6 (–2.8, –0.4)		0.72 (0.55, 0.9)	
Tried smoking (n=3673)	2181	1299 (59.6)	1492		640 (42.9)	*16.7 (14, 19.4)* ^d^		1.96 (1.74, 2.18)	
Tried alcohol (n=3699)	2201	1903 (86.5)	1498		1136 (75.8)	*10.6 (8.5, 12.8)* ^d^		2.04 (1.75, 2.33)	
Tried other substances (n=3633)	2161	294 (13.6)	1472		122 (8.3)	5.3 (3.6, 7)		1.75 (1.44, 2.09)	

^a^Note that the total number of respondents for each factor differs due to missing data.

^b^HDI: highest density interval.

^c^OR: odds ratio.

^d^Estimated differences that, with 95% probability, are above the prespecified cutoff for practical equivalence are italicized.

^e^ADHD: attention deficit hyperactivity disorder.

^f^ASD: autism spectrum disorder.

## Discussion

### Principal Findings

Using a large and representative sample of Swedish adolescent pupils, we found that excessive smartphone use was more prevalent among girls (approximately 60% of all respondents) than among boys (approximately 35% of all respondents). Furthermore, excessive smartphone use was robustly associated with a substantially higher prevalence of poor sleep and, with slight differences between grades and gender, with higher frequencies of having tried smoking, alcohol, and other substances. Among girls, both in the 9th grade of primary school and 2nd grade of secondary school, we found that excessive smartphone use was robustly associated with a higher frequency of often feeling low and feeling anxious. Several other factors differed reliably from zero between the groups, although these differences did not, with 95% probability, exceed the ROPE. Our study adds to the knowledge of excessive smartphone use by investigating the corresponding male and female characteristics and possible associated factors among adolescents in an ordinary Swedish school setting.

Excessive smartphone users of both male and female genders in the 9th grade showed a disproportionate high prevalence of having used cigarettes and alcohol. A similar observation was made for smartphone users of the 2nd grade of secondary school, but in this grade, boys also had a higher probability of experience with illicit drugs. Similar results can be found in the literature; for example, Marmet et al [12] investigated the coexistence of behavioral and substance addiction among adult men and found that individuals with smartphone addiction were more likely to also be addicted to alcohol, tobacco, and illicit drugs. Behavioral and substance addiction have previously been reported as heavily related, and a sharing of a common personality trait has been hypothesized. Our findings warrant for additional research on excessive smartphone use in adolescents in order to implement prevention plans to hinder the development of other forms of addiction [3,27].

The relationship between ADHD and excessive smartphone use has been previously established [1,2,28,29]. The mechanism is thought to act through the lack of social interactions with others, a key characteristic in patients with ADHD, who concordantly feel a stronger need to be assured by and connected to others. Another suggested mechanism is the tendency to be easily bored typically exhibited by individuals with ADHD, resulting in a search for constant stimulation [30-32]. Children with ASD spend significantly more time using screen-based media than any leisure activity, and the correlation between internet addiction and ASD has already been established [15,16,33-37]. In ASD, the mechanism of internet overuse is considered to be due to their autistic traits: restricted, repetitive patterns of behavior, interests, or activities [38]. Some studies prove that children with ASD can learn via smartphone use, especially when the content is responsive to their interests, which makes smartphone use a valuable experience.

In this study, we found a relationship between ADHD and excessive use of smartphone with the strongest probability among boys in the 2nd grade of high school, but it did not exceed the ROPE in any of the groups. We also found increased probability of excessive smartphone use in individuals with ASD, which was the strongest among boys in the 9th grade of primary school but the probability did not exceed the ROPE in any of the groups. One possible explanation is that ADHD and ASD were self-reported; even though the questionnaire was filled in anonymously, one still cannot rule out the tendency to underreport stigmatizing diagnoses as ADHD and ASD.

In none of the groups, loneliness was associated with excessive smartphone use. Previous research suggests a reversed relationship in which close relationships serve as a protective factor against smartphone addiction, when investigating a population comprising both boys and girls [39]. Perhaps our finding could be considered in correspondence with findings that girls, unlike boys, usually use their phones for social reasons such as social media or texting; hence, they may not express a feeling of loneliness [39]. The act of ignoring others in favor of smartphone use at a social setting, also called phone snubbing (or *phubbing*), has become increasingly common. This is associated with poorer relationship satisfaction and lower family well-being and can be supported by other psychological effects in relation to the increased use of electronic devices, such as feeling low or anxious [40-42]—a finding we were able to verify in our study.

In both age groups, we found that girls who reported excessive smartphone use had a higher probability of often feeling low and often feeling anxious. This finding is in line with previous research findings stating excessive smartphone use is significantly associated with depression and anxiety [43,44].

Elhai et al [10] performed a systematic review on problematic smartphone use and reported that both anxiety and depression are related to problematic smartphone use. The female gender is usually described as a risk factor for problematic smartphone use [10], but whether the female gender also increases the negative consequences thereof, such as psychological complaints, is a question for future studies to answer.

Furthermore, the reporting of less than 7 hours of sleep per night (labeled as “poor sleep” in this study) was reported in both sexes and in both grades. Standard sleep recommendations for teenagers (14-17 years) propose 8 to 10 hours of sleep on a daily basis [45,46]. The importance of sleep during adolescence is a key factor for many neurobiological processes, and sleep contributes to physical and mental health [47,48]. Over the past 20 years, sleep patterns among adolescents have changed, and a link to the increasing amount of time adolescents of today spend on the internet has been suggested [47,49,50]. Royant-Parola et al [51] found that smartphone use, in particular, is associated with poor sleep and negative daily functioning, as well as negative mood. The use of screens such as smartphones and sleep patterns have been previously studied, and suggested proposed mechanisms include (1) displacement of time spent sleeping by time spent using screens, (2) psychological stimulation from screen media content, and (3) alerting and circadian effects of exposure to light from screens [52]. Many adolescents use their smartphones just before bedtime, often leaving their phones in bed and repeatedly and frequently checking for notifications. This behavior is thought to increase smartphone use over time, engaging the person in social reassurance from friends and partners and increasing the possibility for excessive smartphone use [53]. Billieux et al [54,55] described this type of behavior is associated with depression and anxiety. This is also in line with our findings, since participants with the highest probability of poor sleep (ie, girls in both age groups) also had the highest probability of feeling low and feeling anxious.

### Strengths and Limitations

This study has some limitations. One of the limitations is the cross-sectional design of the study, which does not allow for conclusions to be drawn regarding causation since such this would require a longitudinal investigation. Moreover, all the measures used for this study were based on self-report, which implies a risk for recall bias that could influence the findings. One could also argue for the use of more objective measures, such as electronic registration of smartphone use, as well as more objective indicators of psychological health (eg, cortisol profiles and actual diagnoses).

This study also has considerable strengths. These include the large, representative sample size along with the high response rate, which reduces the risk of selection bias. The survey also included many variables that are not to be found in registers and can only be captured in questionnaires or interviews. Another strength is the Bayesian approach to statistical analysis, which facilitates genuine probabilistic statements about our findings. Furthermore, using the ROPE procedure as a guide to determine the effects that may be of clinical and practical importance offer further robustness to our findings. Future research exploring excessive smartphone use during adolescence should use longitudinal design for an in-depth understanding of the topic.

### Conclusions

Although results varied across gender and grade in terms of robustness and size of the estimated difference, overall, we found that excessive smartphone use was associated with a higher frequency of multiple suspected associated factors, including ever having tried smoking, alcohol, and other substances; poor sleep; and often feeling low and often feeling anxious. Moreover, our findings suggest that girls with excessive smartphone use are more prone to experience psychological health concerns than boys—a discrepancy that warrants further investigation. The current study brings light to some features and distinctions of a relevant potentially problematic behavior among adolescents of today.

## Appendix

Multimedia Appendix 1 Supplementary material.

## Abbreviations

ADD: attention deficit disorder
ADHD: attention deficit hyperactivity disorder
ASD: autism spectrum disorder
HDI: highest density interval
OR: odds ratio
ROPE: region of practical equivalence
