# Supplementary material for: Gender-Based Differences and Associated Factors Surrounding Excessive Smartphone Use Among Adolescents: Cross-sectional Study
Source: JMIR Pediatr Parent. 2021 Nov 22;4(4):e30889. doi: 10.2196/30889 (PMC8663478; doi:10.2196/30889)
Supplement: Multimedia Appendix 1 [file pediatrics_v4i4e30889_app1.docx]

**APPENDIX**

The present study is based on data from the ***Health survey of Children and Young People in Skåne 2016 - a study of children’s and young people’s living conditions, living habits and health***.

**Parental letter**

In a parental letter the families were informed beforehand of the survey’s purpose and conduct. It was clearly stated that participation was voluntary, that the parent or guardian could notify the school or teacher beforehand if they did not want their child to participate, and that every student of course had the right to abstain from answering the questionnaire and could make this decision at the time when it was conducted at school. It was also clearly stated that the questionnaire would be answered anonymously and that no individual student would be recognizable in the presentation of results as all data would be presented at an aggregated level (municipal and regional).

**Questionnaire**

The questions below are from the 26-page questionnaire directed to students in 9th grade of primary school and 2th grade of secondary school.

INTRODUCTION

Hello!

We would be grateful if you could complete this survey which is aimed at schoolchildren in Skåne.

The questionnaire includes issues such as school, leisure, family, security, activities, and tobacco and alcohol habits. The survey is conducted by Region Skåne, in collaboration with Kommunförbundet Skåne.

It is very important that you and your fellow students complete the survey. By doing so, you give valuable insights about how children and young people experience life today and the results can be used in various ways to improve your daily life. The results will also be used to conduct research on the links between living conditions, living habits, social factors and health among children and young people. It is important that you answer honestly and that you do not talk to others while you fill in the form. If you are not comfortable answering a question, just leave it blank.

**Participating in this survey is voluntary and your answers will be treated confidentially.**

HERE ARE SOME QUESTIONS ABOUT YOU AND YOUR FAMILY

**A1 My biological sex is …..**

Male

Female

HERE ARE SOME QUESTIONS ABOUT YOUR HEALTH

**B1 How would you describe your health in general?**

Very good

Good

Fairly good

Bad

Very bad

**B4 How often, within the last 6 months, have you suffered from the following?**

*(Mark each alternative with a cross)*

Almost More Once a Once a Hardly

every than week month ever or

day once a week never

Headache

Stomachache

Backache

Buzzing in your ears/Tinnitus

**Feeling low**

Feeling irritable/ bad tempered

**Feeling anxious/ worried**

Difficulties in getting to sleep

Dizziness

**B7 On weekdays, how many hours per night do you usually sleep?**

Less than 7 hours

7-9 hours

More than 9 hours

**B8 Do you presently have a close friend with whom you can talk in confidence about almost any personal matter?**

I have no close friends

I have one close friend

I have two close friends

I have more than two close friends

**B14 Do you have any long-term illness or disability?**

*(Mark each alternative with a cross)*

Yes No

Impaired hearing

Impaired eyesight impossible to correct

by glasses or contact lenses

Physical disability

Reading and writing disabilities, dyslexia

**ADHD or ADD**

Other disability

Long-term illness (e.g. diabetes
allergy, epilepsy)

Mental retardation

**Autism / Asperger's Syndrome**

HERE ARE SOME QUESTIONS ON YOUR RECREATIONAL HABITS

**D5 How well do the following statements apply to you?**

(The term "phone" includes use of smartphones and iPads/tablets in the following statements)

*(Mark each alternative with a cross)*

Very Quite Not Almost Never

often often often never

I have felt bothered when I could not check my phone

I have caught myself doing things on my phone

that I was not really interested in

I have felt a strong need to check my phone to see if

anything new has happened

I have spent less time than I should with either family,

friends or doing schoolwork due to my phone

I find myself using my phone even in places/situations

where it is not appropriate

I have tried unsuccessfully to spend less time using

my phone

HERE ARE SOME QUESTIONS ABOUT ALCOHOL

*By alcohol we mean beer, medium / strong beer, strong cider, alcopops, wine, fortified wine and spirits. Even homemade and smuggled spirits and spirits contained in drinks or shots count.*

*Do not count alcoholic beverages below 2.8%, e.g. light beer or light cider.*

**F1a Have you ever drunk alcohol?**

Yes

No Continue to question F11

HERE ARE SOME QUESTIONS ABOUT SMOKING AND SNUFF

**G1a Do you smoke cigarettes?**

No, I have never smoked

No, but I have tried

**How many cigarettes do you smoke per day?**

I smoke
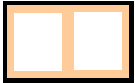
 cigarettes per day

No, I have smoked but gave it up

Yes, every day

Yes, almost every day

Yes, at parties

Yes, occasionally

HERE ARE SOME QUESTIONS ABOUT DRUGS

*By drugs we mean hashish, marijuana, Spice, amphetamines, ecstasy, LSD, cocaine, heroin, GHB, or the like.*

**H3a Have you ever used narcotics?**

*(You can mark more than one alternative)*

Yes, during the past 30 days

Yes, during the past 12 months

Yes, more than 12 months ago

No Continue to question H6
